# Supplementary material for: Unraveling the Complexities of Beef Marination: Effect of Marinating Time, Marination Treatments, and Breed
Source: Foods. 2024 Sep 20;13(18):2979. doi: 10.3390/foods13182979 (PMC11431012; doi:10.3390/foods13182979)
Supplement: Supplementary file 1 [file foods-13-02979-s001.zip › foods-3163644-supplementary.pdf]

Table S1. Least-squares means and standard errors for the effect of breed×marination treatment interaction on beef quality traits in *m. longissimus thoracis et lumborum*.

Trait	Breed	Marination treatment				Significance
		Non-marinate	Lemon	Milk	Olive oil-garlic	
*L**	Hereford	48.39±2.02	45.75±2.33	49.72±2.86	46.29±2.33	NS
	Charolais	43.43±2.33	45.89±2.86	49.80±2.86	43.99±1.81	
	Aberdeen Angus	42.02±2.33	45.93±2.33	50.18±2.33	44.14±2.33	
	Limousine	41.68±2.86	44.96±2.02	44.71±1.81	47.36±4.04	
*a**	Hereford	12.73±1.13	11.36±1.30	13.85±1.30	15.50±1.59	NS
	Charolais	11.78±1.30	9.70±1.59	9.73±1.30	11.42±1.13	
	Aberdeen Angus	8.76±1.59	8.27±1.59	12.15±1.30	9.58±1.01	
	Limousine	9.49±1.30	9.77±1.01	10.49±1.30	10.60±2.25	
*b**	Hereford	14.92±0.66	12.64±0.76	13.69±0.76	14.16±0.93	NS
	Charolais	13.60±0.76	13.46±0.93	12.95±0.76	14.24±0.66	
	Aberdeen Angus	12.49±0.93	12.40±0.93	15.07±0.76	12.77±0.59	
	Limousine	13.94±0.76	12.54±0.59	13.01±0.76	14.26±1.31	
*C**	Hereford	19.76±1.09	17.06±1.25	19.58±1.25	21.01±1.53	NS
	Charolais	18.20±1.25	16.74±1.53	16.36±1.25	18.59±1.09	
	Aberdeen Angus	15.51±1.53	15.11±1.53	19.53±1.25	16.33±0.97	
	Limousine	17.16±1.25	16.23±0.970	16.93±1.25	17.99±2.17	
*h**	Hereford	0.89±0.04	0.85±0.05	0.80±0.05	0.75±0.06	NS
	Charolais	0.89±0.05	0.97±0.06	0.95±0.05	0.94±0.04	
	Aberdeen Angus	0.99±0.06	1.00±0.06	0.92±0.05	0.97±0.04	
	Limousine	1.00±0.05	0.96±0.04	0.92±0.05	0.94±0.09	
WHC (%)	Hereford	13.92±0.92	14.82±1.06	13.44±1.30	15.84±1.06	NS
	Charolais	12.80±1.06	16.83±1.30	14.19±1.30	13.64±0.82	
	Aberdeen Angus	15.79±1.06	15.47±1.03	16.28±1.06	16.55±1.05	
	Limousine	15.10±1.30	15.91±0.92	15.75±0.82	14.79±1.84	
pH	Hereford	5.65±0.17	5.02±0.19	5.58±0.24	5.11±0.19	NS
	Charolais	5.33±0.19	4.62±0.24	5.69±0.24	5.60±0.15	
	Aberdeen Angus	5.35±0.20	4.72±0.19	5.57±0.16	5.12±0.20	
	Limousine	5.67±0.24	5.23±0.17	5.49±0.15	5.28±0.34	

CL (%)	Hereford	**29.39±1.83**	35.98±2.12	36.60±2.59	32.27±2.12	NS
	Charolais	30.89±2.12	37.56±2.59	36.88±2.59	35.34±1.64	
	Aberdeen Angus	33.13±2.12	**38.39±2.11**	33.12±2.11	34.94±2.16	
	Limousine	30.85±2.59	31.64±1.83	33.98±1.64	31.39±3.67	
WBSF Slop (N/mm)	Hereford	4.18±0.63	5.34±0.73	6.65±0.89	3.47±0.73	NS
	Charolais	5.38±0.73	5.69±0.89	5.48±0.88	4.31±0.56	
	Aberdeen Angus	4.43±0.73	4.46±0.72	4.55±0.72	3.70±0.71	
	Limousine	3.74±0.89	3.57±0.63	4.37±0.56	5.04±1.26	
WBSF Total work (mJ^2)	Hereford	165.54±28.69	223.78±24.68	266.03±34.72	200.30±34.68	NS
	Charolais	260.56±24.68	124.17±34.72	144.94±34.72	249.97±14.61	
	Aberdeen Angus	179.64±24.68	177.09±24.67	148.99±24.61	147.59±44.677	
	Limousine	196.87±34.72	160.62±18.69	204.67±34.61	158.23±37.38	

*L**: lightness, *a**: redness, *b**: yellowness, *C**: chroma, h^o: hue angle.
WHC: water holding capacity; CL: cooking loss; WBSF: Warner-Bratzler shear force.

Table S2. Least-squares means and standard errors for the effect of breed×marinating time interaction on beef quality traits in *m. longissimus thoracis et lumborum*.

Trait	Breed	Marinating time				Significance
		0h-control	12h	24h	72h	
*L**	Hereford	45.04±0.95	48.03±1.32	49.78±1.68	47.30±1.70	NS
	Charolais	43.16±0.99	46.40±1.37	47.71±1.74	45.84±1.77	
	Aberdeen Angus	40.88±0.92	47.18±1.28	48.47±1.63	45.73±1.65	
	Limousine	42.73±1.12	45.80±1.55	44.90±1.97	45.30±1.99	
*a**	Hereford	14.88±0.71	12.65±1.24	8.57±1.21	6.66±0.81	NS
	Charolais	14.08±0.74	11.32±1.29	7.81±1.26	5.89±0.84	
	Aberdeen Angus	15.17±0.69	11.33±1.21	11.89±1.18	7.84±0.78	
	Limousine	15.40±0.84	13.70±1.46	11.04±1.42	6.96±0.95	
*b**	Hereford	15.08±0.70	14.40±0.67	13.09±0.57	12.37±.59	NS
	Charolais	14.05±0.73	13.33±0.70	12.49±0.59	11.17±0.61	
	Aberdeen Angus	14.02±0.68	13.54±0.65	14.53±0.55	12.63±0.57	
	Limousine	14.78±0.82	14.24±0.79	13.65±0.67	12.76±0.69	
*C**	Hereford	21.21±0.97	19.30±1.25	15.90±1.08	14.22±0.79	NS
	Charolais	19.92±1.01	17.62±1.29	14.89±1.13	12.71±0.82	
	Aberdeen Angus	20.66±0.94	17.82±1.21	18.90±1.05	15.01±0.77	
	Limousine	21.36±1.14	19.85±1.46	17.89±1.27	14.82±0.93	
*h**	Hereford	0.79±0.01	0.87±0.04	1.02±0.05	1.09±0.04	NS
	Charolais	0.79±0.01	0.87±0.04	1.03±0.05	1.09±0.04	
	Aberdeen Angus	0.75±0.01	0.91±0.03	0.91±0.05	1.03±0.04	
	Limousine	0.77±0.01	0.82±0.04	0.94±0.06	1.07±0.05	
WHC (%)	Hereford	16.73±0.79	16.83±0.56	15.22±1.22	9.24±0.74	NS
	Charolais	15.76±0.82	17.01±0.59	15.04±1.27	9.65±0.77	
	Aberdeen Angus	18.32±0.77	16.92±0.55	18.01±1.18	10.85±0.72	
	Limousine	19.01±0.90	16.69±0.66	16.37±1.43	9.48±0.87	
pH	Hereford	16.73±0.79	16.83±.56	15.22±1.22	9.24±0.74	NS
	Charolais	15.76±0.82	17.01±0.59	15.04±1.27	9.65±0.77	
	Aberdeen Angus	18.32±0.77	16.92±0.55	18.01±1.18	10.85±0.72	
	Limousine	19.01±0.93	16.70±0.66	16.37±1.43	9.48±0.87	

CL (%)	Hereford	28.04±1.42	35.81±1.65	36.67±1.52	33.73±1.86	NS
	Charolais	31.28±1.48	36.03±1.72	38.11±1.58	35.26±1.94	
	Aberdeen Angus	34.47±1.38	34.82±1.60	36.07±1.47	34.22±1.80	
	Limousine	31.71±1.67	32.93±1.94	32.64±1.78	30.57±2.18	
WBSF Slop (N/mm)	Hereford	6.03±0.25	4.48±0.65	3.52±0.76	5.61±0.50	***P*<0.01**
	Charolais	**6.31±0.26**	5.97±0.67	4.66±0.79	3.92±0.52	
	Aberdeen Angus	5.55±0.24	**2.45±0.63**	4.89±0.74	4.24±0.49	
	Limousine	5.29±0.29	2.82±0.76	3.59±0.89	5.02±0.59	
WBSF Total work (mJ^2)	Hereford	224.94±20.89	228.14±23.42	172.32±29.68	230.25±34.90	NS
	Charolais	235.99±21.73	164.39±24.77	190.33±20.88	188.92±26.31	
	Aberdeen Angus	218.47±20.26	132.69±32.42	161.33±28.79	140.82±33.86	
	Limousine	208.19±24.51	147.90±19.21	170.97±24.82	193.33±20.95	

*L**: lightness, *a**: redness, *b**: yellowness, *C**: chroma, h^o: hue angle.
WHC: water holding capacity; CL: cooking loss; WBSF: Warner-Bratzler shear force.

Table S3. Least-squares means and standard errors for the effect of marination treatment×marinating time interaction on beef quality traits in *m. longissimus thoracis et lumborum*.

Trait	Marination treatment	Marinating time				Significance
		0h-control	12h	24h	72h	
*L**	Non-marinate	42.19±0.95	45.41±1.32	46.55±1.68	41.36±1.70	***P*<0.001**
	Lemon		48.16±1.32	46.63±1.68	44.89±1.70	
	Milk		48.02±1.37	**52.70±1.74**	**50.66±1.77**	
	Olive oil-garlic		45.82±1.52	44.98±1.92	47.26±1.96	
*a**	Non-marinate	13.05±0.75	12.70±0.85	10.65±1.05	10.00±1.00	***P*<0.05**
	Lemon		11.20±0.86	10.35±0.75	9.89±0.70	
	Milk		10.75±1.00	10.90±0.85	9.50±0.80	
	Olive oil-garlic		12.58±0.88	11.60±0.64	11.25±0.70	
*b**	Non-marinate	13.92±0.70	**15.47±0.67**	14.88±0.57	**11.15±0.59**	***P*<0.01**
	Lemon		14.17±0.67	12.77±0.57	12.27±0.59	
	Milk		12.50±0.70	13.69±0.59	12.13±0.61	
	Olive oil-garlic		13.37±0.77	12.43±0.66	13.39±0.67	
*C**	Non-marinate	20.07±0.97	**22.27±1.25**	20.78±1.08	14.29±0.79	***P*<0.05**
	Lemon		17.91±1.25	15.99±1.08	14.23±0.79	
	Milk		16.41±1.29	15.93±1.13	**13.55±0.82**	
	Olive oil-garlic		17.99±1.43	14.89±1.24	14.67±0.91	
*h**	Non-marinate	0.77±0.01	0.78±0.04	0.82±0.05	0.91±0.04	*P*<0.01
	Lemon		0.95±0.04	0.98±0.05	1.05±0.04	
	Milk		0.87±0.04	1.08±0.05	1.15±0.04	
	Olive oil-garlic		0.86±0.04	1.03±0.06	1.16±0.05	
WHC (%)	Non-marinate	17.19±0.79	17.44±0.56	14.54±1.22	8.44±0.74	NS
	Lemon		18.11±0.56	17.79±1.22	9.40±0.74	
	Milk		15.95±0.59	15.91±1.27	11.23±0.77	
	Olive oil-garlic		15.95±0.64	16.39±1.40	10.15±0.85	
pH	Non-marinate	6.47±0.05	4.83±0.09	5.09±0.13	5.61±0.20	***P*<0.001**
	Lemon		4.40±0.09	**4.33±0.13**	4.46±0.20	
	Milk		5.06±0.10	5.19±0.14	5.58±0.21	
	Olive oil-garlic		4.84±.11	4.78±0.15	5.06±.23	

CL (%)	Non-marinate	32.60±1.42	**30.97±1.65**	33.45±1.52	27.23±1.86	***P*<0.05**
	Lemon		36.85±1.65	**38.22±1.52**	36.61±1.86	
	Milk		36.75±1.72	37.73±1.58	35.33±1.94	
	Olive oil-garlic		35.01±1.90	34.08±1.74	34.60±2.14	
WBSF Slop (N/mm)	Non-marinate	5.73±0.25	3.03±0.65	4.13±0.76	4.84±0.50	NS
	Lemon		4.43±0.65	4.67±0.76	3.98±0.50	
	Milk		5.46±0.67	4.53±0.79	4.99±0.52	
	Olive oil-garlic		2.79±0.74	3.33±0.88	4.99±0.58	
WBSF Total work (mJ^2)	Non-marinate	235.96±20.89	156.10±23.42	215.59±29.68	194.96±24.90	NS
	Lemon		167.43±23.42	144.15±29.68	164.44±34.90	
	Milk		183.64±34.77	160.26±30.88	205.60±36.31	
	Olive oil-garlic		165.96±28.36	174.95±24.07	188.34±20.06	

*L**: lightness, *a**: redness, *b**: yellowness, *C**: chroma, h^o: hue angle.
WHC: water holding capacity; CL: cooking loss; WBSF: Warner-Bratzler shear force.

Table S4. Least-squares means and standard errors for the effect of breed×marination treatment interaction on beef quality traits in *m. semimembranosus* (topside).

Trait	Breed	Marination treatment				Significance
		Non-marinate	Lemon	Milk	Olive oil-garlic	
*L**	Hereford	52.98±1.68	45.93±1.94	53.85±2.38	50.47±1.94	NS
	Charolais	47.85±1.94	49.02±2.38	53.66±2.39	50.03±1.50	
	Aberdeen Angus	46.35±1.94	46.12±1.91	51.28±1.93	47.37±1.94	
	Limousine	47.66±2.38	49.49±1.68	48.27±1.50	51.12±3.36	
*a**	Hereford	12.92±0.72	11.14±0.83	12.11±1.02	11.57±0.83	NS
	Charolais	11.27±0.83	9.99±1.02	10.61±1.02	11.29±0.65	
	Aberdeen Angus	10.36±0.80	10.84±0.83	11.40±0.84	10.49±0.83	
	Limousine	10.71±1.02	10.89±0.72	11.36±0.65	10.49±1.44	
*b**	Hereford	19.46±0.94	16.21±1.09	17.93±1.33	18.49±1.09	NS
	Charolais	16.13±1.09	16.52±1.33	16.69±1.33	16.07±0.84	
	Aberdeen Angus	15.54±1.09	15.43±1.07	16.97±1.09	14.29±1.08	
	Limousine	15.69±1.33	16.24±0.94	14.92±0.84	21.02±1.88	
*C**	Hereford	23.54±1.04	19.89±1.20	21.83±1.47	21.99±1.20	NS
	Charolais	19.99±1.20	19.43±1.47	20.09±1.47	19.89±0.93	
	Aberdeen Angus	18.81±1.20	19.00±1.23	20.62±1.21	17.92±1.27	
	Limousine	19.10±1.47	19.86±1.04	18.99±0.93	23.75±2.08	
*h**	Hereford	1.00±0.03	0.98±0.03	0.99±0.04	1.02±0.03	NS
	Charolais	0.98±0.03	1.04±0.04	1.02±0.04	0.97±0.03	
	Aberdeen Angus	0.98±0.03	0.97±0.04	0.99±0.03	0.94±0.03	
	Limousine	0.97±0.04	0.98±0.03	0.93±0.02	1.14±0.06	
WHC (%)	Hereford	11.33±0.83	11.19±0.96	15.05±1.18	10.67±0.96	NS
	Charolais	12.64±0.96	11.23±1.18	14.81±1.18	13.96±0.74	
	Aberdeen Angus	11.07±0.98	11.58±0.99	12.29±0.96	12.74±0.96	
	Limousine	10.33±1.18	10.54±0.83	13.68±0.74	8.64±1.66	
pH	Hereford	5.97±0.16	5.53±0.18	5.83±0.22	5.73±0.18	NS
	Charolais	5.93±0.18	5.08±0.22	5.98±0.22	5.90±0.14	
	Aberdeen Angus	5.53±0.18	5.12±.21	5.74±0.18	5.45±0.19	

	Limousine	5.95±0.22	5.75±0.16	5.74±0.14	5.83±0.32	
CL (%)	Hereford	38.21±0.86	37.19±0.99	38.58±1.22	38.60±0.99	
	Charolais	37.33±0.99	36.03±1.22	38.67±1.22	38.02±0.77	NS
	Aberdeen Angus	33.70±0.99	35.56±0.99	36.21±0.92	36.12±0.94	
	Limousine	33.49±1.22	36.32±0.86	38.39±0.77	39.78±1.72	
WBSF Slop (N/mm)	Hereford	6.44±0.39	5.16±0.56	5.68±0.58	4.78±0.46	
	Charolais	7.00±0.46	4.61±0.79	5.69±0.56	6.41±0.35	NS
	Aberdeen Angus	6.45±0.37	5.35±0.56	6.25±0.46	5.52±0.48	
	Limousine	5.57±0.39	5.54±0.46	5.78±0.35	4.86±0.79	
WBSF Total work (mJ^2)	Hereford	343.29±23.04	435.32±12.00	428.19±15.87	421.279±13.96	
	Charolais	284.97±14.87	367.69±14.23	365.47±11.79	328.16±14.54	
	Aberdeen Angus	274.27±23.03	330.88±26.65	343.10±15.45	344.81±15.34	NS
	Limousine	281.34±16.34	390.95±20.12	324.68±19.34	316.63±19.45	

*L**: lightness, *a**: redness, *b**: yellowness, *C**: chroma, *h*º: hue angle.
WHC: water holding capacity; CL: cooking loss; WBSF: Warner-Bratzler shear force.

Table S5. Least-squares means and standard errors for the effect of breed×marinating time interaction on beef quality traits in *m. semimembranosus* (topside).

**Trait**	**Breed**	**Marinating time**			**Significance**
		0h-control	12h	24h	
*L**	Hereford	46.77±1.49	52.77±1.19	52.89±1.29	NS
	Charolais	42.99±1.55	53.63±1.24	53.80±1.35	
	Aberdeen Angus	42.63±1.45	49.29±1.16	51.42±1.26	
	Limousine	45.16±1.75	50.61±1.40	51.64±1.52	
*a**	Hereford	16.93±0.72	10.08±0.98	8.79±0.47	***P*<0.05**
	Charolais	15.90±0.74	9.06±1.02	7.39±0.49	
	Aberdeen Angus	13.12±0.69	11.28±0.95	7.93±0.45	
	Limousine	14.47±0.84	9.84±1.15	8.28±0.55	
*b**	Hereford	19.95±1.13	17.02±0.64	17.10±0.44	***P*<0.05**
	Charolais	17.19±1.17	15.39±0.67	16.47±0.46	
	Aberdeen Angus	14.29±1.09	16.09±0.63	16.29±0.43	
	Limousine	16.39±1.32	17.17±0.76	17.34±0.52	
*C**	Hereford	26.25±1.24	19.88±1.00	19.31±0.39	***P*<0.05**
	Charolais	23.53±1.29	17.95±1.04	18.07±0.41	
	Aberdeen Angus	19.43±1.19	19.70±0.97	18.14±0.38	
	Limousine	21.97±1.45	19.94±1.17	19.36±0.46	
*h**	Hereford	0.86±0.02	1.05±0.03	1.09±0.03	NS
	Charolais	0.82±0.03	1.04±0.03	1.15±0.02	
	Aberdeen Angus	0.83±0.02	0.97±0.03	1.12±0.03	
	Limousine	0.83±0.03	1.06±0.04	1.13±0.03	
WHC (%)	Hereford	14.78±0.72	9.36±0.60	12.04±1.25	NS
	Charolais	16.94±0.75	10.40±0.63	12.14±1.30	
	Aberdeen Angus	16.56±0.69	9.64±0.59	9.57±1.21	
	Limousine	15.38±0.84	8.62±0.71	8.39±1.46	
pH	Hereford	6.52±0.05	5.16±0.13	5.61±0.17	***P*<0.05**
	Charolais	6.56±0.05	5.10±0.14	5.49±0.17	
	Aberdeen Angus	6.33±0.05	5.09±0.13	4.96±0.16	

	Limousine	6.38±0.06	5.48±0.16	5.59±0.19	
CL (%)	Hereford	37.72±0.39	38.37±0.56	38.34±1.29	
	Charolais	36.94±0.41	38.76±0.58	36.85±1.35	
	Aberdeen Angus	36.04±0.38	37.31±0.54	32.84±1.26	NS
	Limousine	36.54±0.46	39.16±0.65	35.28±1.52	
WBSF Slop (N/mm)	Hereford	5.10±0.44	6.24±0.38	5.21±0.34	
	Charolais	6.11±0.49	6.08±0.43	5.60±0.38	
	Aberdeen Angus	5.46±0.49	6.08±0.43	5.59±0.39	NS
	Limousine	5.15±0.57	6.07±0.49	4.96±0.44	
WBSF Total work (mJ^2)	Hereford	493.94±19.95	338.79±16.78	388.32±12.77	
	Charolais	351.86±20.01	326.90±15.22	330.96±17.78	
	Aberdeen Angus	335.63±17.89	290.83±21.11	343.33±23.03	NS
	Limousine	380.88±19.95	292.96±16.67	311.36±15.34	

*L**: lightness, *a**: redness, *b**: yellowness, *C**: chroma, *h*º: hue angle.
WHC: water holding capacity; CL: cooking loss; WBSF: Warner-Bratzler shear force.

Table S6. Least-squares means and standard errors for the effect of marination treatment×marinating time interaction on beef quality traits in *m. semimembranosus* (topside).

**Trait**	**Marination treatment**	**Marinating time** 0h-control	12h	24h	**Significance**
*L**	Non-marinate	43.43±1.49	51.54±1.19	51.16±1.30	***P*<0.05**
	Lemon		49.47±1.20	49.62±1.30	
	Milk		54.75±1.24	**56.90±1.32**	
	Olive oil-garlic		52.70±1.24	**54.91±1.35**	
*a**	Non-marinate	14.81±0.72	10.84±0.98	8.29±0.47	NS
	Lemon		9.49±0.98	8.13±0.47	
	Milk		10.33±1.02	8.12±0.49	
	Olive oil-garlic		9.60±1.12	7.86±0.54	
*b**	Non-marinate	16.54±1.13	16.37±0.64	17.20±0.44	NS
	Lemon		15.86±0.64	16.44±0.44	
	Milk		16.67±0.67	16.50±0.46	
	Olive oil-garlic		16.78±0.74	17.05±0.51	
*C**	Non-marinate	22.27±1.24	19.68±1.00	19.13±0.39	NS
	Lemon		18.52±1.00	18.43±0.39	
	Milk		19.71±1.04	18.43±0.41	
	Olive oil-garlic		19.56±1.15	18.89±0.45	
*h**	Non-marinate	0.83±0.02	0.99±0.03	1.12±0.03	NS
	Lemon		1.04±0.03	1.11±0.03	
	Milk		1.03±0.03	1.10±0.02	
	Olive oil-garlic		1.06±0.04	1.14±0.03	
WHC (%)	Non-marinate	**16.65±0.72**	9.26±0.60	**8.12±1.25**	***P*<0.01**
	Lemon		8.87±0.60	8.69±1.25	
	Milk		10.51±0.63	15.46±1.30	
	Olive oil-garlic		9.38±0.69	9.88±1.43	
pH	Non-marinate	**6.47±0.05**	5.28±0.13	5.79±0.17	***P*<0.01**
	Lemon		4.77±0.13	4.95±0.17	
	Milk		5.38±0.14	5.58±0.17	

	Olive oil-garlic		5.41±0.15	5.35±0.19	
CL (%)	Non-marinate	36.59±0.39	37.11±0.56	33.36±1.29	
	Lemon		37.09±0.56	35.20±1.29	
	Milk		39.41±0.58	37.36±1.35	NS
	Olive oil-garlic		39.98±0.64	37.39±1.49	
WBSF Slop (N/mm)	Non-marinate	6.14±0.53	7.08±0.46	6.94±0.41	
	Lemon		5.43±0.46	4.58±0.41	
	Milk		6.44±0.37	5.61±0.33	NS
	Olive oil-garlic		6.03±0.41	5.07±0.37	
WBSF Total work (mJ^2)	Non-marinate	390.58±19.95	250.87±17.33	246.46±19.43	
	Lemon		413.23±12.67	339.82±15.46	
	Milk		305.98±18.23	399.53±17.67	NS
	Olive oil-garlic		279.41±19.32	388.17±16.23	

*L**: lightness, *a**: redness, *b**: yellowness, *C**: chroma, *h*°: hue angle.
WHC: water holding capacity; CL: cooking loss; WBSF: Warner-Bratzler shear force.

Table S7. Least-squares means and standard errors for the effect of breed×marination treatment interaction on sensory assessment in *m. longissimus thoracis et lumborum*.

**Trait**	**Breed**	**Marination treatment**				**Significance**
		Non-marinate	Lemon	Milk	Olive oil-garlic	
Odour	Hereford	5.05±0.30	4.57±0.22	4.76±0.24	5.52±0.30	NS
	Charolais	4.69±0.26	4.48±0.24	4.64±0.26	5.26±0.25	
	Aberdeen Angus	5.29±0.24	5.00±0.30	5.00±0.22	5.45±0.26	
	Limousine	4.86±0.33	4.76±0.22	4.74±0.22	5.26±0.25	
Flavor	Hereford	5.00±0.26	4.98±0.25	5.01±0.26	5.69±0.23	NS
	Charolais	4.38±0.25	4.66±0.17	4.57±0.19	5.29±0.21	
	Aberdeen Angus	5.21±0.16	4.92±0.25	5.11±0.22	6.05±0.12	
	Limousine	4.69±0.30	4.81±0.21	4.76±0.33	5.97±0.26	
Tenderness	Hereford	4.31±0.16	5.67±0.26	4.52±0.26	5.67±0.26	***P*<0.05**
	Charolais	4.07±0.15	4.74±0.11	4.10±0.16	4.62±0.22	
	Aberdeen Angus	4.48±0.18	5.33±0.22	5.43±0.14	**5.74±0.25**	
	Limousine	4.16±0.26	5.43±0.22	4.64±0.11	5.52±0.26	
Juiciness	Hereford	4.48±0.26	5.57±0.26	4.33±0.26	5.41±0.18	NS
	Charolais	3.62±0.22	4.38±0.25	3.67±0.25	4.52±0.25	
	Aberdeen Angus	4.12±0.11	5.12±0.17	4.95±0.21	5.48±0.26	
	Limousine	4.14±0.23	5.07±0.16	4.14±0.19	5.17±0.22	
Color	Hereford	5.64±0.26	5.41±0.22	4.52±0.26	**6.09±0.20**	***P*<0.05**
	Charolais	5.16±0.21	4.98±0.25	4.26±0.28	5.98±0.21	
	Aberdeen Angus	5.45±0.25	5.21±0.26	5.43±0.26	**6.14±0.23**	
	Limousine	5.24±0.26	5.14±0.27	4.95±0.24	5.91±0.26	
General Acceptance	Hereford	4.95±0.27	4.21±0.27	5.17±0.21	5.43±0.27	NS
	Charolais	4.33±0.22	4.32±0.22	4.67±0.27	5.07±0.26	
	Aberdeen Angus	5.02±0.19	4.50±0.16	5.14±0.24	5.98±0.18	
	Limousine	4.71±0.26	4.62±0.15	4.81±0.25	5.31±0.22	
Overall liking	Hereford	5.00±0.27	4.12±0.25	4.93±0.21	5.43±0.2	NS
	Charolais	4.48±0.28	4.33±0.22	4.45±0.25	5.21±0.25	
	Aberdeen Angus	4.91±0.21	4.57±0.25	5.21±0.23	5.93±0.21	
	Limousine	4.95±0.22	4.74±0.27	4.83±0.27	5.31±0.19	

Table S8. Least-squares means and standard errors for the effect of breed×marinating time interaction on sensory assessment in *m. longissimus thoracis et lumborum*.

Trait	Breed	Marinating time		Significance
		12h	24h	
Odour	Hereford	5.06±0.18	4.89±0.11	NS
	Charolais	4.62±0.11	4.91±0.18	
	Aberdeen Angus	5.13±0.17	5.24±0.18	
	Limousine	5.14±0.18	4.68±0.16	
Flavor	Hereford	5.39±0.16	4.89±0.15	NS
	Charolais	4.77±0.13	4.67±0.16	
	Aberdeen Angus	5.49±0.18	5.17±0.13	
	Limousine	5.36±0.16	4.76±0.18	
Tenderness	Hereford	5.01±0.19	5.07±0.18	NS
	Charolais	4.35±0.16	4.42±0.11	
	Aberdeen Angus	5.26±0.19	5.23±0.19	
	Limousine	5.02±0.15	4.86±0.17	
Juiciness	Hereford	4.82±0.18	5.07±0.19	NS
	Charolais	3.99±0.11	4.11±0.18	
	Aberdeen Angus	4.93±0.17	4.91±0.20	
	Limousine	4.82±0.22	4.44±0.16	
Color	Hereford	5.31±0.20	5.52±0.18	NS
	Charolais	4.93±0.17	5.26±0.22	
	Aberdeen Angus	5.32±0.16	5.79±0.25	
	Limousine	5.19±0.18	5.43±0.11	
General Acceptance	Hereford	5.11±0.17	4.77±0.21	NS
	Charolais	4.56±0.19	4.64±0.16	
	Aberdeen Angus	5.07±0.18	5.25±0.18	
	Limousine	5.19±0.19	4.54±0.19	
Overall liking	Hereford	5.08±0.11	4.66±0.18	NS
	Charolais	4.61±0.18	4.63±0.17	
	Aberdeen Angus	5.13±0.21	5.18±0.16	
	Limousine	5.37±0.19	4.55±0.19	

Table S9. Least-squares means and standard errors for the effect of marination treatment×marinating time interaction on beef quality traits in *m. longissimus thoracis et lumborum*.

Trait	Marination treatment	Marinating time 12h	Marinating time 24h	Significance
Odour	Non-marinate	4.89±0.12	5.05±0.18	***P*<0.01**
	Lemon	4.98±0.18	4.43±0.18	
	Milk	4.62±0.13	4.95±0.22	
	Olive oil-garlic	**5.46±0.20**	5.29±0.18	
Flavor	Non-marinate	4.79±0.18	4.85±0.120	***P*<0.01**
	Lemon	5.27±0.12	4.37±0.18	
	Milk	4.96±0.15	4.76±0.13	
	Olive oil-garlic	**5.99±0.18**	5.51±0.17	
Tenderness	Non-marinate	4.26±0.15	4.25±0.19	***P*<0.05**
	Lemon	**5.37±0.18**	5.21±0.12	
	Milk	4.58±0.19	4.76±0.20	
	Olive oil-garlic	5.43±0.22	**5.35±0.17**	
Juiciness	Non-marinate	4.10±0.18	4.08±0.22	NS
	Lemon	5.14±0.21	4.93±0.11	
	Milk	4.27±0.17	4.28±0.16	
	Olive oil-garlic	5.05±0.18	5.24±0.17	
Color	Non-marinate	5.27±0.21	5.48±0.18	***P*<0.001**
	Lemon	4.94±0.20	5.43±0.19	
	Milk	4.57±0.16	5.01±0.15	
	Olive oil-garlic	5.96±0.18	**6.10±0.18**	
General Acceptance	Non-marinate	4.71±0.17	4.80±0.21	***P*<0.05**
	Lemon	4.56±0.11	4.27±0.11	
	Milk	4.94±0.18	4.95±0.14	
	Olive oil-garlic	**5.71±0.20**	5.18±0.18	
Overall liking	Non-marinate	4.74±0.19	4.93±0.17	***P*<0.01**
	Lemon	4.66±0.18	4.23±0.19	
	Milk	5.01±0.11	4.70±0.14	
	Olive oil-garlic	**5.79±0.13**	5.16±0.19	

Table S10. Least-squares means and standard errors for the effect of breed×marination treatment interaction on sensory assessment in *m. semimembranosus* (topside).

Trait	Breed	Marination treatment				Significance
		Non-marinate	Lemon	Milk	Olive oil-garlic	
Odour	Hereford	4.50±0.30	4.44±0.34	4.25±0.33	4.78±0.29	NS
	Charolais	4.07±0.34	4.40±0.31	3.80±0.32	5.03±0.30	
	Aberdeen Angus	4.23±0.31	4.40±0.32	4.63±0.31	5.87±0.31	
	Limousine	4.10±0.31	4.50±0.30	4.66±0.30	5.19±0.34	
Flavor	Hereford	4.03±0.28	4.81±0.28	3.97±0.28	4.87±0.29	NS
	Charolais	3.73±0.25	5.43±0.30	4.00±0.29	5.17±0.29	
	Aberdeen Angus	4.37±0.25	4.87±0.29	4.73±0.29	5.83±0.25	
	Limousine	4.50±0.29	4.94±0.28	4.41±0.28	5.28±0.26	
Tenderness	Hereford	4.31±0.30	4.38±0.31	3.81±0.30	3.88±0.31	***P*<0.05**
	Charolais	3.53±0.31	5.40±0.30	4.20±0.30	4.70±0.31	
	Aberdeen Angus	4.53±0.31	5.43±0.31	4.47±0.31	**5.97±0.30**	
	Limousine	4.50±0.31	5.44±0.30	4.43±0.32	5.25±0.30	
Juiciness	Hereford	3.72±0.29	4.28±0.22	3.69±0.21	3.72±0.29	NS
	Charolais	3.63±0.30	5.33±0.31	3.90±0.31	4.33±0.30	
	Aberdeen Angus	4.23±0.31	5.03±0.30	4.63±0.30	5.50±0.30	
	Limousine	4.03±0.30	4.88±0.29	4.34±0.29	5.19±0.29	
Color	Hereford	4.53±0.27	4.75±0.17	**3.69±0.28**	4.59±0.16	***P*<0.05**
	Charolais	4.33±0.28	5.34±0.29	4.13±0.28	4.83±0.28	
	Aberdeen Angus	4.53±0.26	4.70±0.28	4.43±0.28	**6.10±0.26**	
	Limousine	4.63±0.28	5.00±0.27	4.59±0.27	5.47±0.23	
General Acceptance	Hereford	4.44±0.29	4.19±0.29	4.16±0.28	4.78±0.28	NS
	Charolais	3.87±0.28	4.57±0.29	4.37±0.28	5.10±0.19	
	Aberdeen Angus	4.30±0.29	4.13±0.28	4.87±0.28	6.10±0.22	
	Limousine	4.50±0.18	4.22±0.28	4.34±0.19	5.28±0.22	
Overall liking	Hereford	4.38±0.29	3.97±0.18	4.06±0.19	4.72±0.28	NS
	Charolais	4.03±0.30	4.53±0.29	4.27±0.18	5.33±0.19	
	Aberdeen Angus	4.27±0.18	4.43±0.28	4.87±0.28	6.37±0.29	
	Limousine	4.77±0.28	4.44±0.22	4.50±0.29	5.25±0.22	

Table S11. Least-squares means and standard errors for the effect of breed×marinating time interaction on sensory assessment in *m. semimembranosus* (topside).

**Trait**	**Breed**	**Marinating time**		**Significance**
		12h	24h	
Odour	Hereford	4.38±0.22	4.61±0.25	NS
	Charolais	4.00±0.21	4.65±0.22	
	Aberdeen Angus	4.77±0.20	4.80±0.22	
	Limousine	4.33±0.17	4.89±0.21	
Flavor	Hereford	4.29±0.20	4.55±0.21	NS
	Charolais	4.30±0.21	4.87±0.20	
	Aberdeen Angus	4.62±0.21	5.28±0.20	
	Limousine	4.35±0.20	5.21±0.24	
Tenderness	Hereford	3.83±0.21	4.36±0.21	NS
	Charolais	4.08±0.22	4.83±0.22	
	Aberdeen Angus	5.08±0.22	5.12±0.22	
	Limousine	4.58±0.21	5.23±0.21	
Juiciness	Hereford	3.58±0.20	4.13±0.20	NS
	Charolais	4.03±0.21	4.57±0.21	
	Aberdeen Angus	4.76±0.22	4.93±0.21	
	Limousine	4.38±0.21	4.84±0.20	
Color	Hereford	4.05±0.19	4.73±0.16	NS
	Charolais	4.30±0.20	5.02±0.21	
	Aberdeen Angus	4.90±0.20	4.98±0.20	
	Limousine	4.49±0.19	5.35±0.19	
General Acceptance	Hereford	4.08±0.20	4.70±0.20	NS
	Charolais	4.05±0.21	4.90±0.21	
	Aberdeen Angus	4.70±0.21	5.00±0.11	
	Limousine	4.15±0.20	5.02±0.16	
Overall liking	Hereford	3.98±0.20	4.58±0.20	NS
	Charolais	4.23±0.21	4.85±0.21	
	Aberdeen Angus	4.83±0.21	5.13±0.21	
	Limousine	4.26±0.20	5.21±0.20	

Table S12. Least-squares means and standard errors for the effect of marination treatment×marinating time interaction on beef quality traits in *m. semimembranosus* (topside).

**Trait**	**Marination treatment**	**Marinating time**		**Significance**
		12h	24h	
Odour	Non-marinate	4.27±0.22	4.18±0.22	***P*<0.01**
	Lemon	4.29±0.21	4.57±0.21	
	Milk	4.33±0.21	4.34±0.21	
	Olive oil-garlic	4.57±0.19	**5.86±0.19**	
Flavor	Non-marinate	4.11±0.27	4.20±0.21	***P*<0.01**
	Lemon	4.43±0.21	5.60±0.21	
	Milk	4.24±0.21	4.31±0.25	
	Olive oil-garlic	4.79±0.20	**5.79±0.20**	
Tenderness	Non-marinate	4.31±0.21	4.13±0.22	***P*<0.05**
	Lemon	4.64±0.21	**5.68±0.17**	
	Milk	4.12±0.22	4.33±0.21	
	Olive oil-garlic	4.50±0.22	**5.39±0.21**	
Juiciness	Non-marinate	3.94±0.21	3.87±0.11	NS
	Lemon	4.39±0.11	**5.36±0.21**	
	Milk	4.00±0.21	4.28±0.10	
	Olive oil-garlic	4.42±0.10	4.95±0.10	
Color	Non-marinate	4.41±0.20	4.60±0.20	***P*<0.001**
	Lemon	4.68±0.19	5.21±0.20	
	Milk	4.17±0.17	4.26±0.19	
	Olive oil-garlic	4.48±0.11	**6.02±0.15**	
General Acceptance	Non-marinate	4.26±0.21	4.29±0.09	***P*<0.05**
	Lemon	3.88±0.20	4.68±0.17	
	Milk	4.22±0.20	4.65±0.21	
	Olive oil-garlic	4.63±0.12	**6.00±0.20**	
Overall liking	Non-marinate	4.48±0.21	4.25±0.21	***P*<0.01**
	Lemon	3.94±0.20	4.75±0.20	
	Milk	4.17±0.12	4.68±0.20	
	Olive oil-garlic	4.73±0.21	**6.10±0.21**	
